# Novel approach to push the limit of temporal resolution in ultrafast electron diffraction accelerators

**DOI:** 10.1038/s41598-022-17453-z

**Published:** 2022-08-03

**Authors:** Beñat Alberdi Esuain, Ji-Gwang Hwang, Axel Neumann, Thorsten Kamps

**Affiliations:** 1 Helmholtz-Zentrum Berlin (HZB), Hahn-Meitner-Platz 1, 14109 Berlin, Germany; 2 Humboldt-Universität zu Berlin Institut für Physik, Newton-Straße 15, 12489 Berlin, Germany

**Keywords:** Ultrafast photonics, Plasma-based accelerators, Ultrafast lasers

## Abstract

Ultrafast electron diffraction techniques that employ relativistic electrons as a probe have been in the spotlight as a key technology for visualizing structural dynamics which take place on a time scale of a few femtoseconds to hundreds femtoseconds. These applications highly demand not only extreme beam quality in 6-D phase space such as a few nanometer transverse emittances and femtosecond duration but also equivalent beam stability. Although these utmost requirements have been demonstrated by a compact setup with a high-gradient electron gun with state-of-the-art laser technologies, this approach is fundamentally restricted by its nature for compressing the electrons in a short distance by a ballistic bunching method. Here, we propose a new methodology that pushes the limit of timing jitter beyond the state-of-the-art by utilizing consecutive RF cavities. This layout already exists in reality for energy recovery linear accelerator demonstrators. Furthermore, the demonstrators are able to provide MHz repetition rates, which are out of reach for most conventional high-gradient electron guns.

## Introduction

Over the last decade, kilometre-long hard X-ray Free-Electron Lasers (FELs)^1–4^ and its follow-up in-depth researches^5–8^ have opened an era for examining ultrafast structural dynamics associated with the diffraction of phase transformations, the making and breaking of bonds in solids, chemical reactions, and rapid biological processes. Recent researches on a semiconductor-based photocathodes^9–11^ have pushed the thermal emittance of electron beams down to few nanometer-radian at femtocoulomb bunch charges, resulting in MeV-class electrons produced by a gun having the transverse coherency close to the hard X-rays. Particularly, the strong scattering power of electrons^12^ enables observation of atomic and molecular structures at a low intensity that can be covered by a cost-effective and compact ultrafast electron diffraction (UED) facility. The MeV-class beam energy not only suppresses background noise from inelastic scattering and velocity mismatch efficiently but also increases penetration depth in a matter and accomplishes sufficient bunch charges at a short bunch length^13^. For instance, a few hundreds of electrons can be packed at a keV energy with a bunch length of 100 fs^14^ which is not enough to produce a diffraction pattern by a single-shot. However, MeV-class electrons effectively overcome this constraint, so up to $10^6$ electrons can be filled in a bunch at the same length. This feature grants the utilization of a pump-probe technique that aims to image the structural changes happening in a sample after an interaction with photons (pump pulse) with a fine scanning of the structure at different time steps attained by adjusting the time-delay between pump and probe pulses. The MeV-UED accelerators have served numerous experiments in condensed media such as phase transitions^15,16^, electron-phonon coupling^17^ and thin film lattice dynamics^18^ for example with pico- and even femtosecond time scales. In the last years, progress has been made regarding the development of various UED techniques for the study of gas phase molecules^19^ and liquid phase samples^20,21^ as well.

One of the confronting challenges that MeV-class UED accelerators still face is the generation of extremely short bunches with consummately reliable stability in terms of transverse emittances and beam arrival time for achieving a high temporal resolution as well as sufficient lateral coherency. For obtaining a bunch length of sub-100 fs at a bunch charge of at least 100 fC, various schemes have been devised^22,23^ and experimentally demonstrated^24^. The fluctuation of elapsed time between pump and probe pulses so-called timing jitter $\tau _{jitter}$, however, overwhelms the temporal distribution of electrons in a bunch, so it determines the temporal resolution of the whole system^25,26^ (see “Methods” for more details). Therefore, in modern UED facilities, the challenge tackled is to achieve an optimum temporal resolution by compromising both contributions as defined by Renkai Li et al.^27^. The existing facilities employ the method in which the phase of radio-frequency (RF) fields in a gun is adjusted to a slight off-crest value for obtaining the minimum bunch length, pushing the performance limit of the timing distribution system^28,29^. However, this approach is still fundamentally limited due to the enhancement of the jitter by setting the off-crest phase and an ultrashort laser pulse is required at the cathode. In the last years, several approaches have been developed to improve the time resolution in MeV UED facilities by compensating the Time-of-Flight (ToF) error using a sophisticated magnetic lattice. Two very recent studies^30,31^ proposed to use a RF gun in combination with a double bend achromatic optic to vanish the ToF jitter and they have demonstrated experimentally a time resolution of sub-50 fs (full-width-at-half-maximum, corresponding to 21.2 fs root-mean-square). In addition, a further study on multiple RF cavities in a beamline was presented by Franssen et al.^32^ for minimizing the arrival time and bunch compression at an initial beam energy of 100 keV. However, this model does not consider the electron beam dynamics in the electron gun, i.e. the emission of low-energy electrons from the cathode, the effects of RF to laser mismatch and the field fluctuations. Furthermore, the space-charge forces, which play a major role in low-energy beams, are neither included, making it difficult to apply in photoinjectors. Here we propose a new methodology to ameliorate the temporal resolution further by implementing additional cavities downstream while also including the complete acceleration process starting from the gun emission together with space charge effects. This scheme allows to free the gun cavity from the burden of a bunch compression and choose the best emission phase for suppressing the mismatch between the laser and RF fields. This shows that the gun itself can reduce the ToF jitter of electrons by a factor of more than 2 respect to initial timing mismatch in the cathode. The subsequent cavities downstream of the gun can increase the efficiency of the compensation by a factor of up to 6. In addition, multiple cavities permit the manipulation of electron distribution in longitudinal phase-space using linearization method based on the stretcher mode^33^. It can reduce bunch length further by compensating for a nonlinear distortion caused by space charge forces. We have obtained a bunch compression factor larger than 40, which corresponds to a bunch length of 22 fs for an initial drive laser pulse length of 1 ps, being this the minimum achievable laser pulse duration in the Sealab facility (see “Methods”). Since the injectors of energy recovery linear accelerators (ERLs) demonstrators (see “Methods”) produce intense electron beams of superior quality in 6-D phase space at an equivalent layout to our idea, this approach offers special scientific opportunities for these facilities (see “Methods” for more details). Our method applies to accelerator facilities with any type of cavities. However, given the capacity of superconducting injectors to operate with one bunch per RF cycle, the utilization of this method in accelerator facilities that involve superconducting cavities not only enables MHz repetition rate UED experiments for highly reversible processes in solids, such as electron plasma dynamics^34^, or even irreversible processes in gas and liquid phase targets, greatly increasing the signal-to-noise ratio, but also enhances the temporal resolution due to the stability provided by the SRF nature. Furthermore, the application of the method can be an important step towards enabling novel technologies that demand ultra-short and stable bunches with a high repetition rate e.g. external injector for plasma-wakefield accelerators^35^.

## Results

### Time-of-Flight Jitter

**Figure 1:**
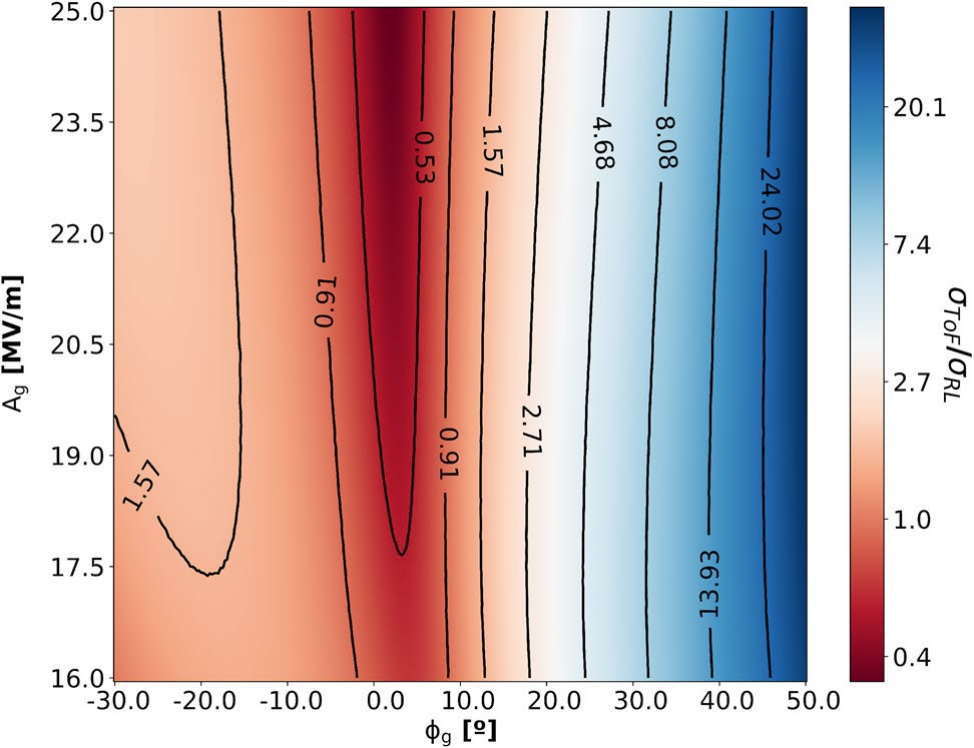
Normalized ToF jitter at the target position as functions of the phase and field gradient of a gun. The 0.0 deg in $x-$axis represents the maximum energy gain in the gun. It is the same convention that is used for the electron emission phase from a cathode in Astra code. The parameter space is determined by achievable parameters for superconducting technology. It is calculated by using Eq. (1) with the partial derivatives $\frac{\partial \Delta t}{\partial A_g}$, $\frac{\partial \Delta t_g}{\partial \phi _g}$, $\frac{\partial E_g}{\partial A_g}$ and $\frac{\partial E_g}{\partial \phi _g}$ that have been emanated by numerical simulations using Astra code.

In UED facilities, the time delay between laser pump and electron probe pulses at a sample is defined by not only the relative arrival timing between two pulses but also fluctuations of the laser arrival time and the Time-of-Flight (ToF) of an electron bunch that are given by $ToF = t_g + t_1$, where $t_g$ denotes the time that electrons need to exit the gun as a function of emission phase and field gradient and $t_1$ is the flight time in a subsequent space. The beam energy which is determined by an amplitude of electric field $E_g$ and phase $\phi _g$ of the gun governs $t_1$. By sharing a laser on the cathode with the pump pulse on a sample, two events of electron ejection from the cathode and laser pulse on the sample become entangled naturally, resulting in the timing jitter $\tau _{jitter}$ being determined solely by the uncertainty of ToF, i.e. $\tau _{jitter} = \sigma _{ToF}$^36^. Fluctuations of the amplitude and phase of RF fields cause beam energy changes that leads to a ToF variation which is inversely proportional to $\gamma ^{3}$, where *gamma* denotes the Lorentz factor. Taking account of fluctuations of laser arrival time on a cathode $\sigma _L$ and amplitude $\sigma _{A_g}$ and phase $\sigma _{\phi _g}$ of the RF field, the total mismatch $\sigma _{RL}$ can be described by $\sigma _{RL} = \sqrt{\sigma _L^2 + \left( \sigma _{\phi _g}/\omega \right) ^2}$. In the subsequent drift, the electron bunch ToF is governed by the energy $E_g$, which is affected by the fluctuation of gun parameters, and the bunch accumulates a further ToF deviation with respect to the design value. The total jitter from the cathode to the end of the drift $L_1$ is given by

1 $$\begin{aligned} \begin{aligned} \sigma _{ToF}^2&= \left( \frac{\partial ToF}{\partial A_g}\right) ^2 \sigma _{A_g}^2 + \left( \frac{\partial ToF}{\partial \phi _g}\right) ^2 \left( \omega \sigma _{RL}\right) ^2 \\&= \left( \frac{\partial t_g}{\partial A_g} - \frac{L_1}{m_0 c^3(\gamma _g^2-1)^{3/2}}\left( \frac{\partial E_g}{\partial A_g} \right) \right) ^2 \sigma _{A_g}^2 + \left( \frac{\partial t_g}{\partial \phi _g} - \frac{L_1}{m_0 c^3(\gamma _g^2-1)^{3/2}}\left( \frac{\partial E_g}{\partial \phi _g} \right) \right) ^2 \left( \omega \sigma _{RL}\right) ^2 , \end{aligned} \end{aligned}$$

where $m_0$ is the rest mass of the electron and *c* is the speed of light. Using Eq. (1), the optimum phase and amplitude of a gun can be investigated. To calculate the partial derivatives $\frac{\partial \Delta t}{\partial A_g}$, $\frac{\partial \Delta t_g}{\partial \phi _g}$, $\frac{\partial E_g}{\partial A_g}$ and $\frac{\partial E_g}{\partial \phi _g}$, numerical simulations are carried out using Astra^37^. The ToF jitter normalized by the initial error $\sigma _{RL}$ at the target position as functions of the phase and field gradient of a gun within achievable parameters for superconducting technology is shown in Fig. 1.

The result highlights three important aspects: firstly, the jitter can be minimized obviously by adjusting emission angles close to the on-crest phase, as it minimizes the bunch energy variation caused by the laser to RF mismatch. Secondly, it is more sensitive to the variation of the phase than the field gradient. Lastly, the higher the gradient, the better for decreasing jitter. For instance, an initial jitter $\sigma _{RL}$ of 318.5 fs can be suppressed down to 138.2 fs with the optimum phase for a field gradient of 20 MV/m. However, for a ballistic bunching scheme that has a phase of − 12.31$^\circ$ to compress the bunch length at about 7 meters downstream, the jitter rises slightly to 452 fs, which is consistent with the previous work^27^ and experimental results at UED facilities^28,30^. This has proven explicitly that extra cavities yield further suppression of the jitter below the minimum value by setting the RF phase to its optimum which is not feasible for a typical UED set-up with an electron gun only. This motivates the investigation of the effect of the cavities for the jitter compensation. The ToF jitter of a beamline composed of a gun and *n*-subsequent cavities with the corresponding drift spaces can be expressed as (see “Methods” for the derivation)

2 $$\begin{aligned} \sigma _{ToF}^2 = \left( \frac{\partial ToF}{\partial A_g}\right) ^2 \sigma _{A_g}^2 + \left( \frac{\partial ToF}{\partial \phi _g}\right) ^2 \left( \omega \sigma _{RL}\right) ^2 + \sum _{i=1}^{n} \left( \left( \frac{\partial ToF}{\partial \phi _i}\right) ^2 \sigma _{\phi _i}^2 + \left( \frac{\partial ToF}{\partial A_i}\right) ^2\sigma _{A_i}^2\right) , \end{aligned}$$

where the $\sigma _{\phi _i}$ and $\sigma _{A_i}$ are the phase and amplitude uncertainties of the i-th cavity, respectively. The partial derivatives of the ToF can be calculated respect to the amplitudes and phases of the cavities in a beamline.

**Figure 2:**
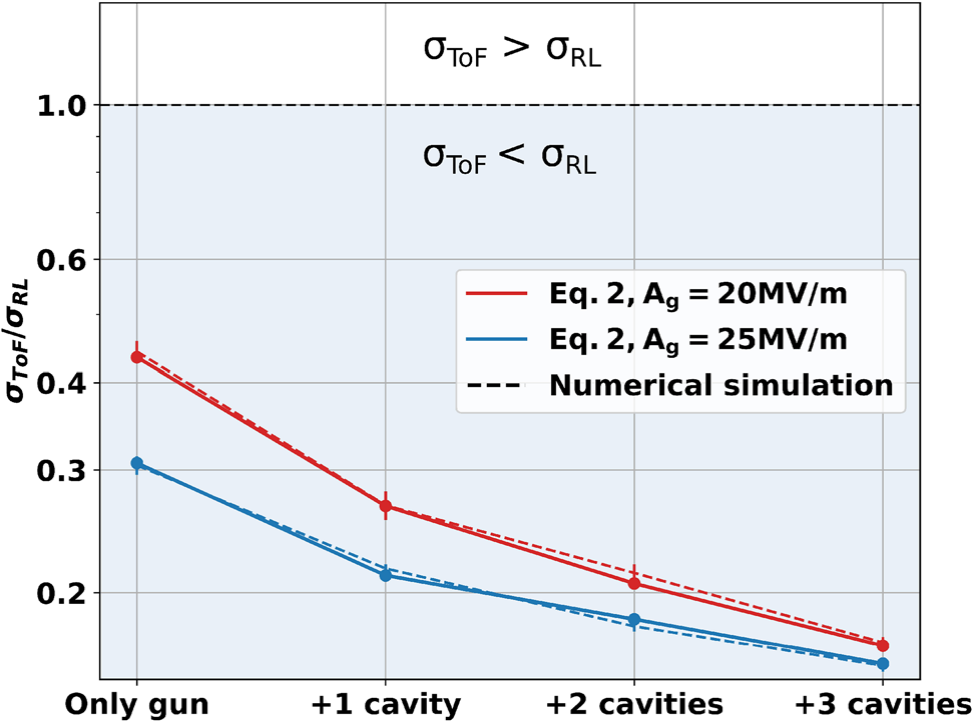
Minimum achievable ToF jitter at the target position with extra cavities downstream of a gun. The calculations are performed with field gradients in the gun of 20 MV/m (red) and 25 MV/m (blue). The solid lines represent the values calculated by using Eq. (2) and the dashed lines represent the simulation results with error bars estimated by analyzing multiple iterations of the tracking simulation and one standard deviation from the result.

The minimum jitters are investigated for the different numbers of cavities numerically and analytically with feasible layout based on Sealab parameters (see “Methods” for more details). The results are listed in Table 1 and shown in Fig. 2. For cases of a gradient of a superconducting electron gun of 20 MV/m and 25 MV/m, the optimum phases and amplitudes of the three successive cavities, as well as the gun, are calculated by using a basin-hopping algorithm^38^.

**Table 1:** Machine parameters for the minimization of ToF jitter in Fig. 2 with an initial jitter of $\sigma _{RL} = {318.5} fs$, cavity phase stability of $\sigma _{\phi } = 0.05^{\circ }$ and amplitude stability of $\sigma _{A} = 1 \times 10^{-4}\, A$.

Gun		Cavity 1		Cavity 2		Cavity 3		$\sigma _{ToF}$ @ 7.64m	
$\mathrm {\phi _g}$ ($^\circ$)	$\mathrm {A_g}$ (MV/m)	$\mathrm {\phi _{1}}$ ($^\circ$)	$\mathrm {A_{1}}$ (MV/m)	$\mathrm {\phi _{2}}$ ($^\circ$)	$\mathrm {A_{2}}$ (MV/m)	$\mathrm {\phi _{3}}$ ($^\circ$)	$\mathrm {A_{3}}$ (MV/m)	Calculated (fs)	Simulated (fs)
2.6	20	X	X	X	X	X	X	**138.4**	**141** ±**5**
3.70	20	$-$ 28.91	8.91	X	X	X	X	**84.8**	**85**±**4**
4.38	20	$-$ 26.40	8.60	$-$ 37.13	7.98	X	X	**65.7**	**68**± **2**
4.95	20	$-$ 17.66	8.91	$-$ 57.01	9.87	$-$ 42.75	9.90	**53.4**	**54**± **1**
1.95	25	X	X	X	X	X	X	**97.7**	**97**±**3**
2.88	25	$-$ 31.54	9.63	X	X	X	X	**67.4**	**69**±**1**
3.73	25	$-$ 16.49	9.84	$-$ 20.67	7.91	X	X	**58.3**	**57**±**1**
3.68	25	$-$ 28.07	7.87	$-$ 8.00	9.88	$-$ 0.03	4.38	**50.3**	**50**±**1**

Significant values are in bold.

Calculated ToF jitter refers to the calculation using Eq. (2) and the simulated one is obtained using Astra.

The results evidence that the three extra cavities, $\mathrm {\left( n = 3 \right) }$, can push the limit of the jitter defined by the synchronization between the laser and RF field reduced by a factor of 5.7. This scheme is widely used in a photoinjector of ERL demonstrators which has a booster module downstream of the gun. The analytical formula in Eq. (2) quantitatively agrees well with the numerical results. As shown in Table 1, the emission phase in the gun has the optimum point close to the on-crest phase to minimize the laser to RF jitter influence in the ToF of the electrons. At the working points that lead to the minimum values, the final arrival time fluctuation is almost immune to $\sigma _{RL}$ and is governed by the amplitude and phase fluctuations in the additional cavities. The compensation by increasing the field gradient in the gun is still effective for the case of none or one cavity available, but the difference diminishes when more cavities are involved.

### Bunch compression with space charge effects

**Figure 3:**
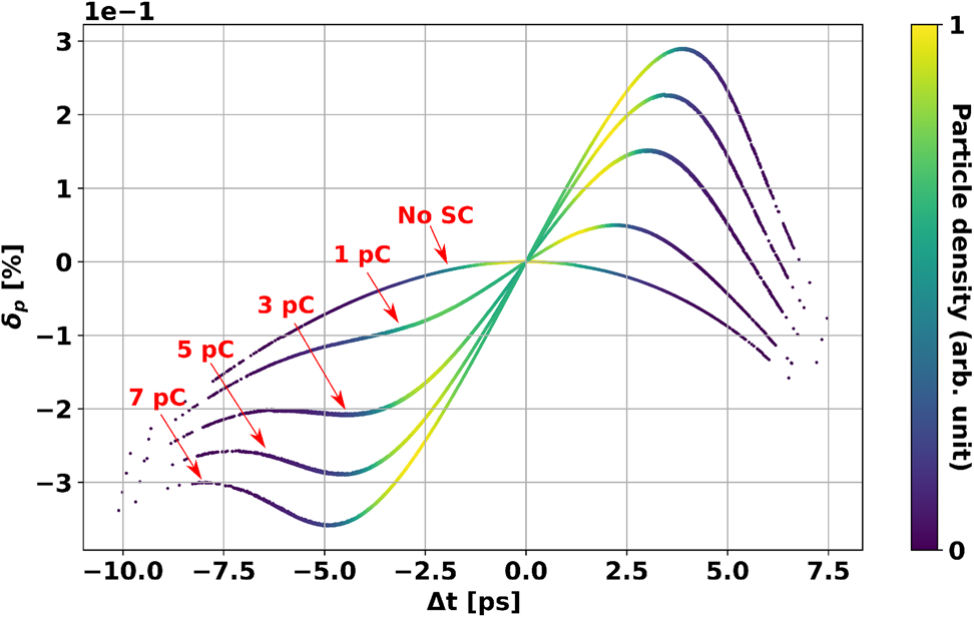
Particle distributions in longitudinal phase-space at the collimator position for various initial bunch charges. The electrons are emitted at on-crest emission phase with a maximum gradient of 20 MV/m, resulting in a kinetic energy of 1.61 MeV. The space-charge induced curvature contribution is added to the RF chirp imprinted in the gun which is clearly visible for the case without space charge. For the higher bunch charges, the split of two distinct cores in the phase space is observed, resulting in the increment of the energy spread and bunch length.

The time resolution in UED machines is not only determined by the ToF jitter but also depends on the temporal distribution of electron beams at a sample (see “Methods” for more details). This does call for the manipulation of electron distribution in longitudinal phase space to achieve a short bunch length at a sample by sacrificing the optimum phase calculated in the above section. Particularly, for the bunch length down to its limit, it is necessary to rectify a nonlinear distortion of the particle distribution in longitudinal phase space which is caused by space charge (SC) forces. The distortion is formed in the first meter after the gun since the transverse phase space of the beam is necessary to be tailored by a collimator with an opening of in the order of a few tens micrometres to acquire its desired transverse emittance, as the minimum achievable emittance without collimation is limited by the minimum laser pulse spot-size of 0.5 mm rms at the cathode^39^. The transverse emittances can be down to suitable value for obtaining a high-quality diffraction pattern, $\epsilon _{x,y} \sim$ 30 nm rad, by selecting a fraction of the electron beams generated by a gun with a bunch charge of a few pC. The SC effect is investigated for the different initial bunch charges and the result is shown in Fig. 3.

**Figure 4:**
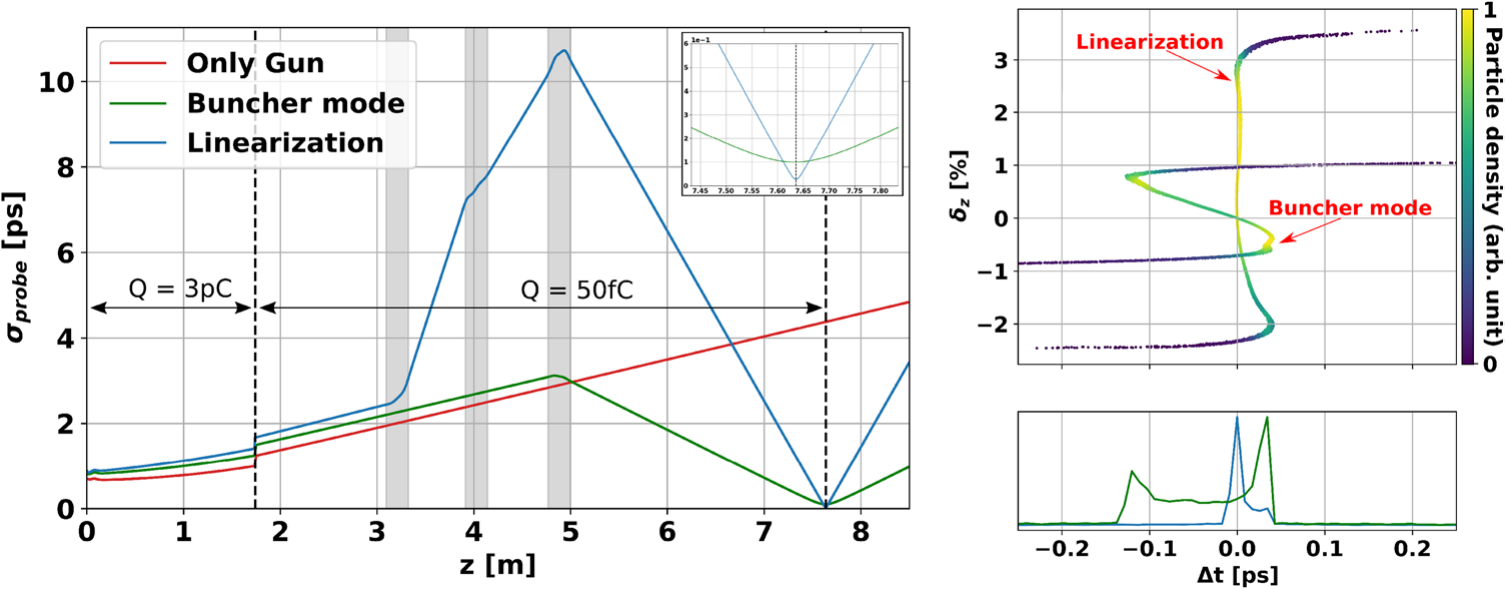
(LHS) Bunch length evolution of an electron bunch with SC forces. The collimator and target positions are indicated with vertical dashed lines. The grey shadowed areas represent the position of the cavities. The initial bunch charge is 3 pC and it is reduced to 50 fC after the collimator. The bunch length for different configurations is shown, always for a gun gradient of 20 MV/m. The red line shows the evolution of the bunch length for only gun, the minimum value at the target is achieved with $\phi _g = -12.3^{\circ }$ for the given initial charge. The green line corresponds to the beamline with one additional cavity in which the emission from the gun happens in on-crest phase. Finally, the blue line shows the bunch length evolution for one of the linearization solutions with three extra cavities, using the same cavity amplitudes and gun phase that minimize the ToF jitter in Table 1. The zoomed image shows the difference between the bunch length at the target for the last two. The final energy is 2.22 MeV for the first two cases and 2.15 MeV for the linearization case. The increase in the rms bunch length at the aperture is given by the radial dependence of the longitudinal particle distribution caused by space charge fields. The outermost particles are collimated, leaving only the closer to on-axis particles which have a larger rms spread. (RHS) On the top side, the phase space of the electron bunch at the aperture position is shown colored according to the particle density for the two cases with shortest bunch length. On the bottom side the longitudinal particle distribution for the two cases is shown. For the linearized solution the maximum compression is limited by the higher order nonlinear distortions.

After the collimator, the bunch charge in the range of 50 fC with the MeV beam energy allows us to ignore the space charge effects downstream. Therefore, the nonlinear distortion only occurs in the section between the cathode and the collimator. The extra cavities downstream can be used to mitigate the effect of the distortion in the bunch compression. The normalized transverse emittance remains almost constant regardless of longitudinal bunch compression. Then, the cavities can contribute not only to reducing the jitter as explained in above section but also to giving adjustable parameters for the longitudinal phase space manipulation. A linearization method is adopted^33^ for rectifying the nonlinear distortion. The main advantage of the method is that the phase-space curvature caused by SC fields can be taken into account in the compensation of the distortion up to the third order. In addition, the linearization allows to use the on-crest emission from the gun to minimize the effect of $\sigma _{RL}$ in the jitter and compress the bunch at the same time. With a linearization method, for the maximum bunch compression with on-crest gun emission, a minimum length of 22 fs at the interaction point can be achieved with an initial laser pulse length of 1 ps, providing a compression factor over 40. This clearly improves the compression that can be achieved with a single buncher cavity under the same emission conditions in the gun. The evolution of the bunch length for the three different schemes can be seen in Fig. 4. The bunch lengths at the target position together with the working points of the RF cavities for each case are shown in Table 2. Moving the target closer to the last cavity would improve the minimum achievable bunch length, but higher gradients are necessary for obtaining the high bunch compression factor. This would also increase the contribution to the ToF jitter given by the amplitude fluctuations. In any case, even if the target is located closer downstream from the last cavity, the shortest bunch length achieved with the buncher mode under the same initial conditions is still a factor 3 longer than the one achieved using the linearization method shown in Fig. 4.

**Table 2:** Working point for the bunch length evolutions in Fig. 4.

Gun		Cavity 1		Cavity 2		Cavity 3		Target @ 7.64 m
$\mathrm {\phi _g}$ ($^\circ$)	$\mathrm {A_g}$ (MV/m)	$\mathrm {\phi _{1}}$ ($^\circ$)	$\mathrm {A_{1}}$ (MV/m)	$\mathrm {\phi _{2}}$ ($^\circ$)	$\mathrm {A_{2}}$ (MV/m)	$\mathrm {\phi _{3}}$ ($^\circ$)	$\mathrm {A_{3}}$ (MV/m)	$\pmb {{\sigma _{ {probe}}}}$ (fs)
$-$ 12.3	20.0	X	X	X	X	X	X	**4377.8**
0.0	20.0	X	X	X	X	$-$ 90.0	6.065	**96.3**
4.95	20.0	129.0	8.91	30.7	9.87	$-$ 110.38	9.92	**21.69**

Significant values are in bold.

**Figure 5:**
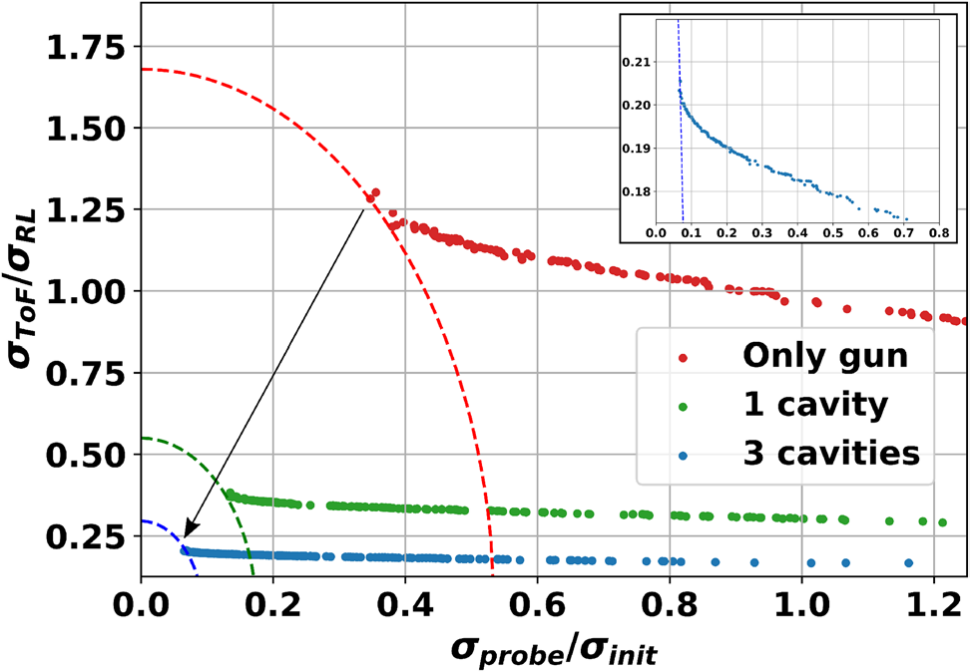
Compromised solutions calculated by multi-objective genetic algorithm^40^ for the optimization of the time resolution in UED accelerators. The red dots represent the pareto-dominant solutions for the beamline with only gun, the green dots represent the solutions for a single additional cavity and the blue dots represent the solutions for the beamline with the three additional cavities. The insert plot shows the zoom of the latest. The dashed lines represent the distance from origin to the closest point for each case, which gives the maximum time-resolution according to Eq. (3). The constrains for the optimization are that the initial laser pulse length at the cathode has to be longer than 1 ps, the initial rms laser spot size larger than 0.5 mm and that the electron bunch at the target must have a charge of at least 50 fC. The pareto fronts show that there is indeed a correlation between bunch compression and ToF jitter, both cannot be minimized simultaneously. For each magnitude the individually achievable minimum values are shown by the asymptotic behaviours of the ends of the pareto fronts, while the curve in between both ends represents the trade-off between them when both are minimized at the same time (better seen in the zoomed-in plot).

The cavity parameters for the longitudinal phase-space curvature compensation, however, are not the same as the optimal working point of the ToF jitter minimization. The linearized bunch configuration results in a ToF jitter of 296.7 fs. It is dominated solely by the field instabilities in the cavities because the effect of $\sigma _{RL}$ has been mitigated by the close to on-crest emission from the gun. Hence, reducing the instabilities directly reduces the ToF jitter. Overall, the correlation between bunch length and ToF jitter presented in^27^ for the only gun case can not be applied for our study since the addition of RF cavities along the beamline makes the analytical description of the bunch compression very challenging. To obtain the optimum time-resolution in UED experiments, compromised solutions are computed using the numerical simulations based on multi-objective genetic algorithm. For the different cases, the pareto-fronts which represent the set of non-dominated solutions, where each objective are considered as equally good are estimated and shown in Fig. 5. The trade-off between compression and jitter can be observed from the result.

For instance, the optimum working point that minimizes the time-resolution while keeping an acceptable lateral coherence length with a final bunch charge of at least 50 fC leads to with a lateral coherence length of 3 nm (for a bunch spot size of 300 mm at the target) and a time resolution of 103 fs (being the last cavity field fluctuations the main contribution with 72 fs) with an initial laser to RF mismatch of 318.5 fs and laser duration of 1.0 ps. The resulting solution is a nearly linearized bunch at the target, with an on-crest emission from the gun. Hence, the scheme with the three cavities reduces the jitter by a factor of about 4 and compresses the bunch in time by a factor slightly above 10 at the same time, while the gun on its own is only able to reduce the bunch length by half without any gain for the jitter compensation. The effect of adding the extra cavities in the beamline improves the time resolution clearly.

## Conclusion

In this study, we have proposed and demonstrated the new approach for improving the time resolution in UED experiments by implementing three additional cavities downstream of a gun, which is widely adopted in an SRF photoinjector of ERL demonstrators. This offers a new strategy to push the limit of the time resolution that has been constrained by state-of-the-art technologies for the ultrashort electron bunch generation from the photocathode and the synchronization between laser and RF cavity. Hence, synchronization between laser and RF fields is no longer the limiting factor in the arrival time jitter and short laser pulses at the cathode in the tens of fs are not required. The compensation of nonlinear distortion caused by space charge effects as well as time-of-flight jitters can be achieved by mastering the phase and gradient of the additional cavities. These milestones are achieved while keeping a large lateral coherence length at a sample for obtaining clear diffraction patterns with a bunch charge of about 100 fC by tailoring the transverse phase-space distribution of a few pC bunches using a collimator with a diameter of a few tens of micrometres. The initial conditions taken into account in the estimations are conservatively assumed, for instance, the maximum gradients on the cavities have been limited to values which are considered easily reachable (see “Methods”), but the real values are expected to be at least 50% higher. The time resolution in the studied method is limited by the field stability of the cavities. The LLRF control is expected to at least match the phase and amplitude instability values used here (see “Methods”), any improvement in the value of the fluctuations would automatically lead to smaller ToF jitter for every studied working point. As an example, reducing the cavity field fluctuations by a factor 2 from $0.05^{\circ }$ to $0.025^{\circ }$ would directly reduce the ToF jitter of the maximum time resolution working point from 91.21 to 43.45 fs, which would improve time resolution from 103 to 63 fs. Reducing the phase instability in the additional cavities even further would directly obviate the need of looking for a trade off between compression and jitter, as the on-crest emission combined with the linearization would directly provide the best achievable time-resolution. The latest LLRF control techniques have already proven cavity phase stability values as low as $0.008^{\circ }$^41^ for a normal conducting 1.3 GHz CW buncher cavity and $<0.01^{\circ }$ for the 1.3 GHz superconducting linac^42^. The application of these values to the additional cavities in the beamline, while keeping the gun phase stability at $0.05^{\circ }$, would increase the time resolution from the linearization solution in Fig. 4 to values below 60 fs. In this scheme, the last cavity contributes mainly to the time resolution with a jitter of 42 fs that can be improved by utilizing an isochronous optics. Further improvements in the phase stability would bring the time resolution to lower values, where the amplitude fluctuations in the cavity fields and the minimum bunch length given by the linearization would become the limiting factors. This highlights the advantage of the approach in this work, the capacity that the additional cavities provide to improve time resolution without the need of improving laser to RF synchronization or reducing the longitudinal electron bunch profile at the cathode. Furthermore, this opens a new opportunity for existing ERL photoinjectors as a new scientific case without significant modifications.

## Methods

### ERL demonstrators

**Figure 6:**
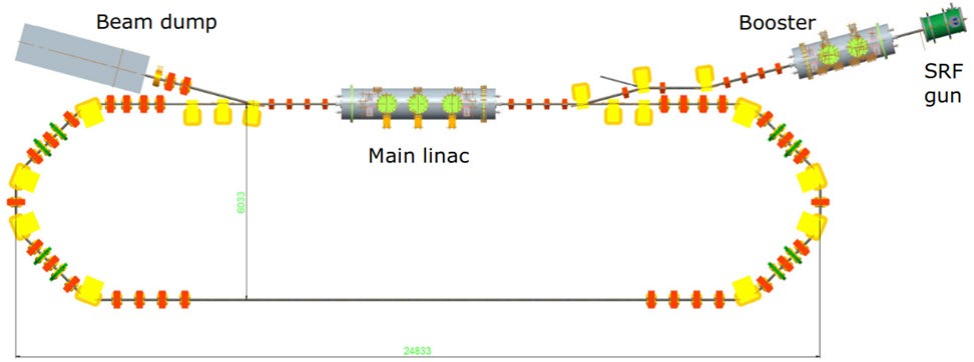
Sketch of the ERL demonstrator in Helmholtz-Zentrum Berlin.

Energy Recovery Linac (ERL) demonstrators are test facilities that aim to verify various physical and technical challenges in the generation, acceleration, transport and energy recovery of high brightness and high average current electron beams in a superconducting radio-frequency (SRF) linear accelerator. During the past decades, several ERL demonstrators have been built in the world^43–45^ and one of them, bERLinPro, is being currently commissioned in Helmholtz-Zentrum Berlin^46^. The layout of the ERL demonstrators follows a common design similar to the bERLinPro shown in Fig. 6. It consists of an injector with a photocathode gun and booster, the main linac, and the recirculator.

These demonstrators have completed the verification of core technologies and the scientific applications of such accelerators are being currently studied^47^. Besides working as test facilities, given the high brilliance and short bunch length that can be achieved, it could be used for Compton back-scattering experiments, THz radiation sources or, as in this case, time-resolved pump-probe experiments.

### Sealab and the SRF photoinjector

**Figure 7:**
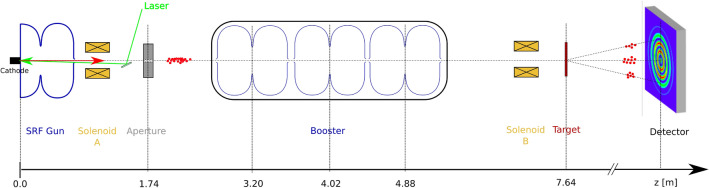
Sketch of the SRF photoinjector beamline with UED related components.

Superconducting Electron Accelerator Laboratory (Sealab) in Helmholtz-Zentrum Berlin is a test bench for beam dynamics, control and instrumentation R &D of high average current, ultrashort and high brightness beams. The main beamline within the Sealab facility is an ERL demonstrator injector. A simplified sketch of the SRF Photoinjector beamline with the UED components can be seen in Fig. 7. The laser system provides green light between 510 and 540 nm and a Gaussian shaped longitudinal profile with rms pulse length in the ps range at the photocathode. The repetition rate of the laser system goes up to 1.3 GHz. The laser spot size at the photocathode is limited in the lower side to 0.5 mm rms radius top-hat distribution. It has a minimum pulse length of 1.0 ps and a Gaussian longitudinal shape. The semiconductor $\mathrm {K_2CsSb}$ photocathode, which operates nominally with an incident wavelength of 520 nm, is located at the back-wall of a superconducting L-band (1.3 GHz) electron gun. The SRF gun operates in continuous wave mode and is designed for a maximum field gradient of 30 MV/m, but the measurements in a test-stand confirm that a gradient of up to 42 MV/m can be achieved in controlled environments. The main beamline elements following the way of the particles downstream the gun are a superconducting solenoid magnet (solenoid A); an aperture plate; three L-band superconducting booster cavities (field gradient up to 20 MV/m); a normal conducting solenoid (solenoid B); a target station for UED samples, located at $\mathrm {z = {7.64}\,{m}}$; and a scintillation-based detector to record the resulting diffraction patterns. For this work, the maximum field gradients in the gun and the additional RF cavities have been conservatively determined to 20 MV/m and 10 MV/m, respectively, to make sure that the used values can be achieved in a beam operation.

The working point of each RF cavity used in the beamline is subjected to fluctuations in amplitude and phase with respect to a reference signal given by a master oscillator. The fluctuations can be controlled by the feedback system of the LLRF control up to a certain point. The superconducting gun cavity has a phase stability $\sigma _{\phi _g} = 0.05^{\circ }$ and an amplitude stability $\sigma _{A_g} = 1 \times 10^{-4}$ A. The added cavities along the beamline have been measured in the test stand to have an improved phase stability of $\sigma _{\phi } = 0.02^{\circ }$. However, for this work, the largest value $\sigma _{\phi } = 0.05^{\circ }$ is adopted for all of them in principle. Furthermore, the laser timing jitter is of 300 fs, which together with the phase instability of the gun results in $\sigma _{RL}={318.5}$ fs.

### Time resolution

The time resolution $R_t$ in a UED experiment defines the capability of discerning minimum temporal extension of the structural dynamics. This quantity can be expressed by a square root of the sum of the quadrature of the different contributions,

3 $$\begin{aligned} R_t^2 = \sigma _{pump}^2 + \sigma _{probe}^2 + \tau _{jitter}^2 + \tau _{vm}^2, \end{aligned}$$

where $\sigma _{pump}$ and $\sigma _{probe}$ stand for the rms length of the laser and electron pulses at a sample, respectively, $\tau _{jitter}$ refers to the fluctuation in elapsed time between the arrival of two pulses, and $\tau _{vm}$ represents the velocity mismatch. Typically, the pump pulse originates from the same laser that illuminates the photocathode to produce electrons. Thus, even if the properties of the laser pulse at two distinct positions are very different, they are inherently synchronized in time. The pump pulse can be converted to a different wavelength via a high harmonic generator and is guided through a delay stage to control the delay between two pulses. Commercially available laser systems can provide a pump pulse length as short as 10 fs. The velocity mismatch $\tau _{vm}$ is given by the time difference originated by the different velocities of photons and electrons in the target material that need to reach the same depths. The quantity causes the elapsed time between the interaction with the photons and with the electrons to differ depending on the depth in the sample. MeV electron beam energies make this contribution to the temporal resolution neglectable compared to lower energy electron beams, in which the velocity mismatch can be the limiting factor^48^.

### ToF jitter in a beamline with multiple RF cavities

**Figure 8:**
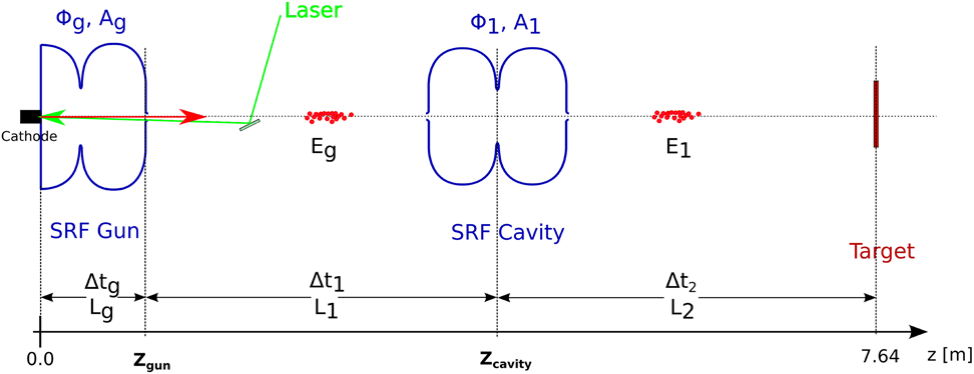
Sketch of the beamline with the elements that affect the time of flight jitter for the case in which only one RF cavity is used between gun and target.

Departing from Eq. (1), the effects of further cavities along the beamline can be modeled using the single-particle approximation. This approximation assumes that the electrons in one bunch receive the same energy acceleration in the cavity, but that, at the same time, their relative position inside the bunch does not change. This approximation is more precise for the high energy bunch. The energy gain in a cavity under this approximation can be interpreted as

4 $$\begin{aligned} \Delta E = A \cos (\phi ) = A \cos (\phi _{0} + \omega \Delta t), \end{aligned}$$

where *A* and $\phi$ represent the amplitude and phase of the cavity respectively. The phase is defined with respect to the phase for the maximum energy gain at the moment in which the electron bunch crosses the cavity. This phase can be related to the ToF of the electron from the cathode to the cavity by the constant $\mathrm {\omega = 2\pi f}$ and the phase $\phi _{0}$ of the RF field at the time $\mathrm {t={0.0}s}$. Figure 8 shows the sketch of the beamline between gun and target with an additional RF cavity in between.

Using the notation in Fig. 8, the total ToF of the electrons is the addition of time needed for the electrons to travel each drift section between the cavities. The beam energy at a given section is determined by all the cavity parameters upstream as

5 $$\begin{aligned} \begin{aligned} E_1&= E_g + A_1 \cos (\phi _{1,0} + \omega \left( \Delta t_g + \Delta t_1 \right) ) \\&= E_g + A_1 \cos \left( \phi _{1,0} + \omega \left( \Delta t_g + \frac{L_1 E_g}{c\left( E_g^2 -m_0 c^2 \right) }\right) \right) . \end{aligned} \end{aligned}$$

Considering that $E_g = E_g(\phi _{g}, A_g)$ and $\Delta t_g = \Delta t_g (\phi _{g}, A_g)$ are extracted from simulations, one can then calculate the ToF jitter as a function of the fluctuation in the cavity parameters $\phi _g, E_g, \phi _{1,0}, E_1$ by error propagation:

6 $$\begin{aligned} \sigma _{ToF}^2 = \left( \frac{\partial ToF}{\partial A_g}\right) ^2 \sigma _{A_g}^2 + \left( \frac{\partial ToF}{\partial \phi _g}\right) ^2 \left( \omega \sigma _{RL}\right) ^2 + \left( \frac{\partial ToF}{\partial \phi _1}\right) ^2 \sigma _{\phi _1}^2 + \left( \frac{\partial ToF}{\partial A_1}\right) ^2\sigma _{A_1}^2 . \end{aligned}$$

The rms error of the cavity parameters are considered to be equal to the gun as they share the same LLRF control system. This same sketch can be directly extended to an arbitrary number of subsequent cavities. The energy after the n-th cavity is given by

7 $$\begin{aligned} E_i = E_g + \sum _{i=1}^{n} A_i \cos \left( \phi _{i,0} + \omega \left( \Delta t_g + \sum _{j=1}^{i}\Delta t_j\right) \right) . \end{aligned}$$

The time the electrons need to reach the end of the beamline is $\mathrm {ToF = \Delta t_g + \sum _{n=1}^{i+1} \Delta t_n}$. The advantage of this approach is that analytical derivatives of the time of flight respect to the resonant cavity parameters can be calculated by deriving Eq. (7). One only needs the initial phases $\phi _{i,0}$ and amplitudes of all the involved cavities together with the simulation results for the ToF and electron bunch energy at the exit of the gun. The final ToF jitter expression for *n*-cavities along the beamline is given in Eq. (2).

### Lateral coherence length

The quality of a diffraction pattern is a combination of sample properties and instrumental factors. The study of favorable sample properties is beyond the scope of this work. The instrumental factors enclose the quality of the electron beam and that of the detection system. The quality of an electron beam for diffraction can be described by its coherence length. The lateral coherence length is a measure of the spatial coherence and is usually defined as:

8 $$\begin{aligned} L_{\perp } = \frac{\lambda }{2\pi \sigma _{\theta }} = \frac{\hslash }{mc} \frac{\sigma _{\perp }}{\epsilon _{n,\perp }}, \end{aligned}$$

where $\lambda = h / \gamma \beta m c$ is the De Broglie wavelength of the electron, $\sigma _{\theta } = \epsilon _{n,\perp } / \gamma \beta \sigma _{\perp }$ is the uncorrelated angular spread, $\sigma _{\perp }$ represents the beam spot size in the transverse directions (*x* or *y*) and $\epsilon _{n,\perp }$ is the normalized transverse emittance in the same direction. The lateral coherence length indicate the maximum distance between atoms/molecules in matter that can produce a diffraction pattern coherently. A criterion for generating a high-quality diffraction pattern in UED accelerators is $L_{\perp } > d_{100}$, i.e. the transverse coherence length should be larger than the largest lattice spacing $d_{100}$ of the sample.

### Numerical simulation

The simulations shown in this work are performed using the Astra^37^ code that can compute macro-particle tracking numerically through user-defined electromagnetic fields with space-charge forces. The initial temporal and spatial distribution is a replica of an incident laser-pulse on a photocathode. The latest response-time measurement of semiconductor photocathodes^49^ shows almost an order of magnitude smaller values than a laser pulse length in SRF Photoinjectors, so this effect is ignored in our calculation. The transverse momentum distribution is chosen so that the normalized transverse emittance of the initial bunch matches the intrinsic emittance of measured for $\mathrm {K_2CsSb}$ photocathodes^39^. The electrons are propagated along the beamline with the simulated field patterns for RF cavities and solenoids. The initial number of macro-particles in the electron bunch is set in order to ensure that at least $1 \times 10^{4}$ macro-particles are still alive after the collimation to mitigate statistical errors. Simulations of Time-of-Flight jitter are performed tracking a reference particle from the cathode to the target with sufficient iterations ($1 \times 10^{4}$). This procedure is repeated for each working point and the rms spread of the time of flight is calculated accordingly. The fluctuations of the field phases and amplitudes in the cavities follows pre-defined Gaussian statistics. For the linearization of the longitudinal phase space using additional cavities, the non-linearity of the collimated beam collimation by using an aperture is estimated by fitting a third order polynomial. The analytical solution for the linearization is found by solving the equations in^33^ with the fitted polynomial. Once an analytical solution is found for the linearization, a particle tracking simulation is conducted to evaluate the space-charge effects. The analytical solution gives an initial working point for an optimizer. Then, numerical optimizations are used to search for the minimum achievable bunch length near the initial condition.

## Data Availability

The data that support the findings of this study are available from the corresponding author upon reasonable request.

## References

[1] Rossbach J (1996). A VUV free electron laser at the TESLA test facility at DESY. Nucl. Instrum. Methods Phys. Res. Sect. A.

[2] Bilderback DH (2010). Energy recovery linac (ERL) coherent hard x-ray sources. New J. Phys..

[3] Kim E-S (2011). Parameter optimizations and performances for the low-charge beams in PAL free-electron laser. IEEE Trans. Nucl. Sci..

[4] de Wijn R (2022). Potential of time-resolved serial femtosecond crystallography using high repetition rate XFEL sources. Appl. Sci..

[5] Amann J (2012). Demonstration of self-seeding in a hard-X-ray free-electron laser. Nat. Photon..

[6] Kang H-S (2017). Hard X-ray free-electron laser with femtosecond-scale timing jitter. Nat. Photon..

[7] Kang H-S (2020). Attosecond XFEL for pump-probe experiments. Nat. Photon..

[8] Nam I (2021). High-brightness self-seeded X-ray free-electron laser covering the 3.5 keV to 14.6 keV range. Nat. Photon..

[9] Karkare S (2014). Ultrabright and ultrafast III-V semiconductor photocathodes. Phys. Rev. Lett..

[10] Cultrera L (2016). Ultra low emittance electron beams from multi-alkali antimonide photocathode operated with infrared light. Appl. Phys. Lett..

[11] Schmeißer M (2018). Towards the operation of Cs-K-Sb photocathodes in superconducting rf photoinjectors. Phys. Rev. Accel. Beams.

[12] Henderson R (1995). The potential and limitations of neutrons, electrons and X-rays for atomic resolution microscopy of unstained biological molecules. Q. Rev. Biophys..

[13] Carbone F (2012). A perspective on novel sources of ultrashort electron and X-ray pulses. Chem. Phys..

[14] Zewail AH (2010). Four-dimensional electron microscopy. Science.

[15] Li J (2022). Direct detection of V–V atom dimerization and rotation dynamic pathways upon ultrafast photoexcitation in $$\rm VO_{2}$$. Phys. Rev. X.

[16] Lu Q (2022). Photoinduced evolution of lattice orthorhombicity and conceivably enhanced ferromagnetism in $${{\rm LaMnO}}_{3}$$ membranes. NPJ Quantum Mater..

[17] Chase T (2016). Ultrafast electron diffraction from non-equilibrium phonons in femtosecond laser heated Au films. Appl. Phys. Lett..

[18] Mannebach EM (2015). Dynamic structural response and deformations of monolayer $$\rm MoS_{2}$$ visualized by femtosecond electron diffraction. Nano Lett..

[19] Shen X (2019). Femtosecond gas-phase mega-electron-volt ultrafast electron diffraction. Struct. Dyn..

[20] Nunes JPF (2020). Liquid-phase mega-electron-volt ultrafast electron diffraction. Struct. Dyn..

[21] Ledbetter K (2020). Photo-dissociation of aqueous $$\rm I_3^{-}$$ observed with liquid-phase ultrafast mega-electron-volt electron diffraction. Struct. Dyn..

[22] Anderson SG (2005). Velocity bunching of high-brightness electron beams. Phys. Rev. Spec. Top. Accel Beams.

[23] Chatelain, R. Radio-frequency pulse compression for high-brightness ultrafast electron diffraction: Design, characterization and application. PhD Thesis, McGill University, Montreal (2014).

[24] Maxson J (2017). Direct measurement of sub-10 fs relativistic electron beams with ultralow emittance. Phys. Rev. Lett..

[25] Chatelain RP (2012). Ultrafast electron diffraction with radio-frequency compressed electron pulses. Appl. Phys. Lett..

[26] Zhu P (2015). Femtosecond time-resolved MeV electron diffraction. New J. Phys..

[27] Li RK (2009). Temporal resolution of MeV ultrafast electron diffraction based on a photocathode RF gun. Nucl. Instrum. Methods Phys. Res. Sect. A.

[28] Weathersby SP (2015). Mega-electron-volt ultrafast electron diffraction at SLAC National Accelerator Laboratory. Rev. Sci. Instrum..

[29] Fu F (2014). High quality single shot ultrafast MeV electron diffraction from a photocathode radio-frequency gun. Rev. Sci. Instrum..

[30] Kim HW (2020). Towards jitter-free ultrafast electron diffraction technology. Nat. Photon..

[31] Qi F (2020). Breaking 50 femtosecond resolution barrier in MeV ultrafast electron diffraction with a double bend achromat compressor. Phys. Rev. Lett..

[32] Franssen JGH (2017). Improving temporal resolution of ultrafast electron diffraction by eliminating arrival time jitter induced by radiofrequency bunch compression cavities. Struct. Dyn..

[33] Zeitler B (2015). Linearization of the longitudinal phase space without higher harmonic field. Phys. Rev. Spec. Top. Accel Beams.

[34] Sun S (2020). Direct imaging of plasma waves using ultrafast electron microscopy. Struct. Dyn..

[35] Wu Y (2021). High-throughput injection-acceleration of electron bunches from a linear accelerator to a laser wakefield accelerator. Nat. Phys..

[36] Yang J (2020). A compact ultrafast electron diffractometer with relativistic femtosecond electron pulses. Quantum Beam Sci..

[37] Flottmann, K. Astra - A Space Charge Tracking Algorithm. https://www.desy.de/~mpyflo (2000).

[38] Wales D (1997). Global optimization by basin-hopping and the lowest energy structures of lennard-jones clusters containing up to 110 atoms. J. Phys. Chem. A.

[39] Bazarov I (2011). Thermal emittance measurements of a cesium potassium antimonide photocathode. Appl. Phys. Lett..

[40] Zitzler E (1999). Multiobjective evolutionary algorithms: A comparative case study and the strength Pareto approach. IEEE Trans. Evol. Comput..

[41] Zhou F (2021). Commissioning of the SLAC Linac Coherent Light Source II electron source. Phys. Rev. Accel. Beams.

[42] Posen S (2022). High gradient performance and quench behavior of a verification cryomodule for a high energy continuous wave linear accelerator. Phys. Rev. Accel. Beams.

[43] Sakanaka S (2018). Construction and commissioning of the compact energy-recovery linac at KEK. Nucl. Instrum. Methods Phys. Res. A.

[44] Angal-Kalinin D (2018). PERLE. Powerful energy recovery linac for experiments. Conceptual design report. J. Phys. G.

[45] Bartnik A (2020). CBETA: First multipass superconducting linear accelerator with energy recovery. Phys. Rev. Lett..

[46] Abo-Bakr, M. *et al.* Status report of berlin energy recovery Linac Project. In *Proceedings of IPAC16*, 4127 (2016).

[47] Kamps, T. *et al*. Scientific opportunies for bERLinPro 2020+, report with ideas and conclusions from bERLinProCamp 2019. arXiv:1910.00881 (arXiv preprint) (2019).

[48] Zhang P (2014). Tilted femtosecond pulses for velocity matching in gas-phase ultrafast electron diffraction. New J. Phys..

[49] Loisch G (2022). Direct measurement of photocathode time response in a high-brightness photoinjector. Appl. Phys. Lett..

